# How Sup35 monomer conformation and amyloid fibril polymorphism determine yeast strain phenotypes

**DOI:** 10.21203/rs.3.rs-7945345/v1

**Published:** 2025-11-03

**Authors:** Motomasa Tanaka, Takashi Nomura, David Boyer, Yusuke Komi, Peng Ge, Rodrigo A Maillard, Piere Rodriguez, Atsushi Yamagata, Mikako Shirouzu, Giuseppe Legname, Bruno Samori, David Eisenberg

**Affiliations:** RIKEN Center for Brain Science; RIKEN Center for Brain Science; University of California Los Angeles; RIKEN Brain Science Institute; University of California, Los Angeles; University of California, Berkeley, QB3 Institute,; University of California; RIKEN; RIKEN; Scuola Internazionale Superiore di Studi Avanzati (SISSA); University of Bologna; UCLA

## Abstract

In the [*PSI*^+^] prion system, the yeast prion protein Sup35 can form structurally distinct amyloid fibrils that lead to distinct transmissible prion states, or strains. However, our understanding of how different Sup35 fibril structures arise and translate to phenotypic variations is limited. Here, using cryo-EM and single-monomer force spectroscopy with optical tweezers, we reveal the structural basis of yeast prion propagation in four wild-type and S17R mutant variants of Sup35 that underlie different [*PSI*^+^] strains. Cryo-EM structures show that the four variants form strikingly distinct fibril structures, which exhibit varying stability and chaperone-accessibility. Force spectroscopy suggests the different distinct fibril structures are derived from distinct monomer conformational ensembles. Further, cryo-EM structures indicate that prion strain strength is correlated with enhanced fibril propagation caused by a combination of low fibril stability and a large separation between the Sup35 fibril core and the Ssa1/Sis1 chaperone-binding region. These results provide a structure-based mechanism for the yeast prion strain phenomenon with implications for understanding amyloid propagation in human neurodegenerative diseases.

## Introduction

Proteins often misfold and form aggregates under cellular stress and senescence. Among protein aggregates, β-sheet rich fibrillar aggregates called amyloid fibrils, are associated with many neurodegenerative diseases. Intriguingly, the same protein can form structurally different fibrils that show distinct phenotypes *in vivo* such as disease severity and brain-region specificity^1–4^. The structure-function correlation observed for distinct amyloid fibrils originating from the same protein has been referred to as the “strain phenomenon”. A growing body of evidence indicates that the strain phenomenon is a universal feature of neurodegenerative diseases, including prion disease, Alzheimer’s disease and Parkinson’s disease^5,6^.

Yeast prions have provided an experimental system for obtaining a better mechanistic understanding of the prion strain phenomenon. It has been shown that the yeast [*PSI*^+^] prion state is caused by the formation of amyloid fibrils of the translation termination factor Sup35. In [*psi*^−^] non-prion yeast, non-amyloid Sup35 causes the ribosome to stop at the premature termination codon in the *ade1-14* nonsense mutation, which prevents cells from producing full-length Ade1, blocking the synthesis of adenine, and leading cells to appear red on YPD medium^7,8^. In the [*PSI*^+^] prion state, Sup35 amyloid loses its translation termination activity, which allows cells to produce full-length Ade1, synthesize adenine, and leading cells to appear white on YPD medium.

**Figure 1:**
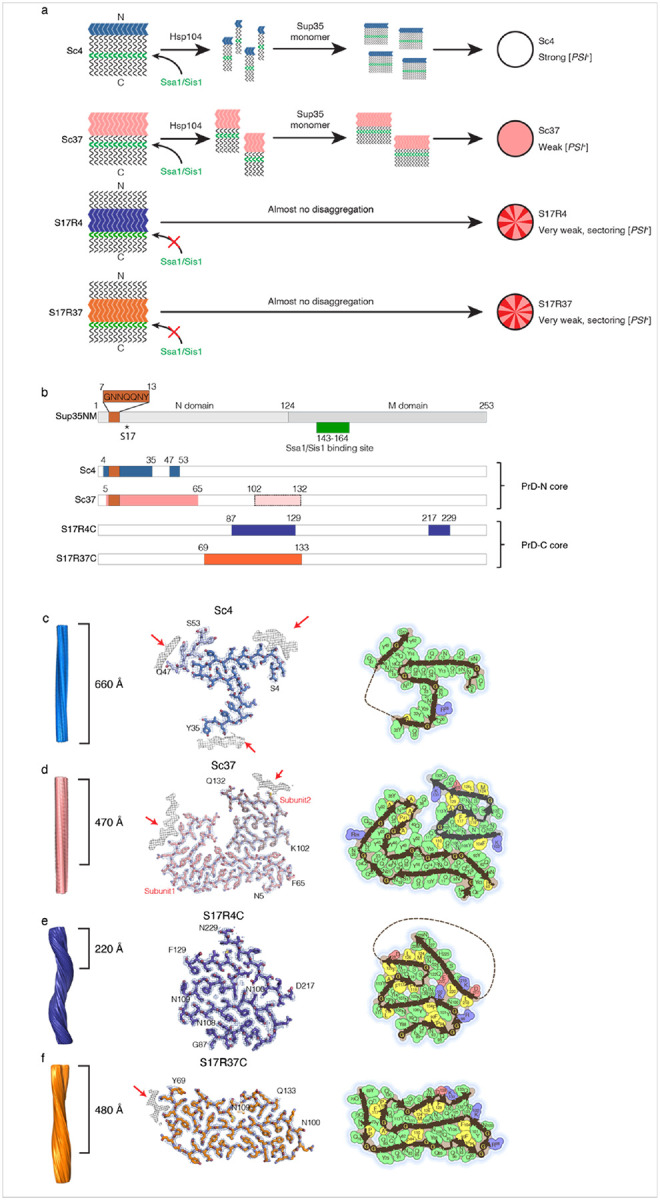
Cryo-EM structure of Sup35NM amyloids **a**, Our previous studies suggest a model in which distinct yeast strain phenotypes are determined by four distinct Sup35NM wild-type and mutant amyloid fibril conformations. **b**, Schematic showing the amyloid core positions. WT Sc4 and Sc37 form N-terminal cores (PrD-N), whereas mutant S17R4C and S17R37C form C-terminal cores (PrD-C). **c–f**, Left: side view showing the fibril axis and the crossover distance of each fibril (**c**, Sc4; **d**, Sc37; **e**, S17R4C; **f**, S17R37C). Middle: atomic model and density map of Sup35NM amyloids. Arrows indicate unidentified electron densities. Right: polarity map at pH 7.4. The polarity maps are colored as follows: hydrophobic in yellow, polar in green, glycine in pink, acidic in red, and basic in blue.

Interestingly, Sup35 can form distinct fibril structures, or polymorphs, that underlie distinct [*PSI*^+^] prion strains (Fig. 1a)^9,10^. Sup35 contains an N-terminal highly aggregation-prone prion domain (N or PrD) and a polar amino acid-rich region (M) (Fig. 1b; M124-D253). The NM domain of Sup35, Sup35NM, forms distinct fibril conformations *in vitro* under different temperatures. Specifically, Sup35NM fibrils formed at 4°C (Sc4) and 37°C (Sc37) display distinct fibril conformations, and when they are introduced into non-prion [*psi*^−^] yeast, they induce strong and weak suppression of the *ade1-14* nonsense mutation, called strong (white) and weak (pink) [*PSI*^+^] strains, respectively (Fig. 1a)^9,11,12^. Previous studies revealed that Sup35 fibril propagation, and thus maintenance of [*PSI*^+^] phenotype, is dependent on the Hsp104/Ssa1/Sis1 chaperone system, which disassembles Sup35 fibrils into seeds^13^. The chaperone-mediated disassembly is crucial for efficient fibril propagation by increasing the number of seeds which can newly recruit monomers into the seeds^7,8^. Further, we have shown that Hsp104/Ssa1/Sis1 chaperone disaggregation activity for Sup35 fibrils is dependent on Ssa1/Sis1 binding to residues T143-K164 in Sup35 (Fig. 1a, b)^14,15^.

To better understand how different Sup35NM fibril conformations lead to distinct yeast phenotypes, the fibril core composition and biophysical characteristics were investigated in different Sup35NM fibrils. Hydrogen/deuterium(H/D)-exchange NMR and mass spectrometry analyses showed that the fibril cores of Sc4 and Sc37 are formed by the N-terminal residues (PrD-N) including S2-A42 and S2-Q72, respectively^11,16^. The Sc4 fibrils, which have a shorter core region compared to Sc37, are more mechanically fragile^12^, and more easily fragmented by the Hsp104/Ssa1/Sis1 chaperone machinery, yielding more fibril fragments^14^. They recruit more endogenous Sup35 protein for aggregation in yeast, leading to the white [*PSI*^+^] phenotype due to the strong suppression of *ade1-14* nonsense mutation (Fig. 1a, top). In contrast, the Sc37 fibrils showed a smaller propensity for chaperone-mediated fragmentation, potentially due to the longer fibril core, resulting in less fibril fragments and less Sup35 aggregation, leading to the pink, weak [*PSI*^+^] phenotype (Fig. 1a, middle top)^14^.

Interestingly, previous studies including yeast genetic screening found that the S17R mutation in Sup35 induces the deep pink, very weak and sectoring [*PSI*^+^] strains^11,17^. Contrary to the N-terminal core of the Sc4 and Sc37 fibrils, NMR and mass spectrometry experiments showed that S17R mutant fibrils formed at 4°C or 37°C (S17R4 and S17R37, respectively) have a core formed by the C-terminal residues of PrD (PrD-C), Q81-L144 and Q62-L144, respectively^11^. The S17R fibrils are highly resistant to chaperone-mediated fragmentation, likely due to the partial burying of the Ssa1/Sis1 chaperone binding region (T143-K164) in the amyloid core^15^. Accordingly, the near complete lack of seed production from the S17R4 and S17R37 fibrils leads to the sectoring, very weak [*PSI*^+^] phenotypes. (Fig. 1a, middle bottom and bottom)

Although our understanding of strains has improved, major unsolved questions remain in prion biology. It has been unclear how the chaperone-binding, fibril stability and amino acid packing synergistically shape and characterize fibril structures and impact yeast prion propagation, leading to distinct [*PSI*^+^] strain phenotypes. The four Sup35NM fibrils and [*PSI*^+^] strains provide an experimental platform suitable for directly investigating the relationship. Importantly, the lack of atomic structures of the four prion strain conformations has hampered our mechanistic understanding of how differences in fibril structures translate into phenotypic variations and why distinct fibrils conformations arise from the same monomeric Sup35NM protein.

To understand the atomic basis of the prion strain phenomenon, we determined cryo-EM structures of Sup35NM Sc4, Sc37, S17R4, and S17R37 fibrils, revealing markedly different fibril core structures with distinct amino acid packing. Furthermore, we performed single-monomer structural analysis via force spectroscopy, which suggests that differences in conformational space of monomer lead to the distinct fibril conformations. Together, we show that both the fibril stability and the extent of exposure of the chaperone-binding region in fibrils are critical determinants of fibril propagation and [*PSI*^+^] phenotypes. These results revealed for the first time the overall relationship between fibril structure and stability, chaperone-mediated fibril fragmentation, fibril propagation, and strain phenotype *in vivo*. More broadly, our findings provide new insights into how monomer and fibril conformations modulate fibril propagation and strain phenotypes.

## Results

### Cryo-EM structures of Sup35NM amyloid fibrils

First, we determined the cryo-EM structure of the Sup35NM wild-type (WT) amyloid which forms at 4°C, termed Sc4, to a resolution of 3.1 Å (Fig. 1b, c and Extended Fig. 1a, and 2a, Table 1). We prepared Sc4 fibrils by polymerizing Sup35NM WT monomer at 4°C with Sc4 seeds (5% mol/mol) (See Methods). The Sc4 amyloid core consists of four β-strands formed by the residues S4–Y35 in the N-terminal region of the prion domain (PrD-N) and the amyloid core region was approximately consistent with the previous observations by the H/D-exchange NMR and mass spectrometry analyses^11,16^. The cryo-EM structure revealed a short β-strand formed by residues Q47–S53, which was not detected in the previous studies^11,16^. Additionally, three other densities resembling β-strands were observed to contact the core (Fig. 1c middle, red arrows), but insufficient electron density prevented their identification at the amino acid level. Notably, unlike the majority of fibril structures, the overall Sc4 amyloid structure lacked extensive intramolecular interactions within each layer of the fiber. Instead, Sc4 fibrils showed intramolecular interactions only in the limited region spanning Q14–Q24. Overall, the Sc4 amyloid structure lacked extensive intramolecular interactions within each layer of the fiber.

Next, we determined the cryo-EM structure of the Sup35NM WT amyloid which forms at 37°C, termed Sc37, to a resolution of 3.1 Å (Fig. 1b, d and Extended Fig. 1b, 2b, Table 1). We prepared Sc37 fibrils by polymerizing Sup35NM WT monomer at 37°C with Sc37 seeds (5% mol/mol) (See Methods). In contrast to the β-strand poor Sc4 structure, the Sc37 amyloid contains 16 β-strands and exhibits a complex folded arrangement of amino acids within each layer, as observed for many other amyloid structures^18^.Furthermore, the Sc37 amyloid core contains a larger number of aromatic amino acids (Y: 14, F: 3) than the Sc4 amyloid core (Y: 7), which are expected to enhance structural stability both within each layer and along the fibril axis through their π-stacking interactions. Notably, the Sc37 amyloid core consists of two major residue ranges: residues N5-P65 (termed subunit 1) and residues K102-Q132 (termed subunit 2). However, while subunit 1 is present in all Sc37 fibrils, 3D classification in RELION showed that among the particles contributing to high-quality reconstructions, half the particles have low subunit 2 occupancy (junk particles, 29%; both subunits 1 and 2, 37%; subunit 1 alone, 34%) (Extended Fig. 3). Interestingly, analysis of classification results revealed that particles with or without subunit 2 are found at an equal frequency within a single Sc37 fibril (see Methods). These results agree well with the mass spectral data showing a protease-sensitive subunit 2 in Sc37 fibrils and a lower protection factor of subunit 2 than that of subunit 1 by H/D-exchange NMR analysis^11,16^.

Third, we determined the cryo-EM structure of the Sup35NM S17R amyloid which forms at 4°C, termed S17R4. We prepared S17R4 fibrils by polymerizing the Sup35NM S17R monomer at 4°C with S17R4 seeds (5% mol/mol) (See Methods). Cryo-EM analysis revealed that S17R4 formed a major polymorph, termed S17R4C, representing 72% of usable particles, and a minor polymorph, termed S17R4N, representing 28% of usable particles (Fig. 1b,e; Extended Fig. 1c-d, 4, 5). S17R4C and S17R4N were determined to resolutions of 2.2 Å and 2.4 Å, respectively (Extended Fig. 2c-d, Table 1). Remarkably, contrary to the PrD-N core of Sc4 and Sc37 fibrils, the core of the S17R4C polymorph was composed of residues Y88-F129 from the C-terminal region of the prion domain (PrD-C) (Fig. 1b,e), representing a drastic change of amyloid core region by the S17R point mutation in Sup35NM in line with previous mass spectrometry data^11^. Additionally, near residues N100-K102 and G122-Q124, a discontinuous β-strand containing approximately 10 residues was observed. Model building experiments allowed us to assign residues D217-N229 to this extra density (See Methods and Extended Fig. 6). Furthermore, 2D and 3D class averages show a strong “fuzzy coat” located on the N-terminal side of the S17R4C fibril core (Extended Fig. 1c, red arrow), suggesting that a semi-folded domain from the highly aggregation-prone N-terminal region may exist in the fuzzy coat. Contrary to S17R4C, the core of the S17R4N polymorph was composed of residues S4-Q38 from the N-terminal region of the prion domain (PrD-N) and the S17R mutation is solvent-exposed in the ordered fibril core (Extended Fig. 5a-b). In addition, a disconnected density belonging to residues N108-A115 forms an interface with Q30-Q38 (Extended Fig. 5b). Interestingly, unlike Sc4, Sc37, and S17R4C fibrils, S17R4N has a right-handed twist (Extended Fig. 5b). Furthermore, S17R4N is notable because of the highly “kinked” conformation of residues N8-Y16, which is reminiscent of low-complexity, aromatic-rich, kinked segments (LARKS) associated with reversible amyloids^19^. S4-N9 have a significant elevation change, forming a spiral-like conformation that crosses almost two layers of the fibril (Extended Fig. 5c).

Finally, we determined the cryo-EM structure of Sup35NM S17R amyloid, which forms at 37°C, termed S17R37 (Fig. 1b,f and Extended Fig. 1e, 2e). We prepared S17R37 fibrils by polymerizing Sup35NM S17R monomer at 37°C with S17R37 seeds (5% mol/mol) (See Methods). Cryo-EM analysis revealed that S17R37 formed a major polymorph, termed S17R37C, representing 70% of usable particles, and a minor polymorph, termed S17R37N, representing 20% of usable particles (Fig. 1b,f; Extended Fig. 1e-f, 7, 8). S17R37C and S17R37N were both determined to resolutions of 2.4 Å (Extended Fig. 2e-f, Table 1). Additionally, two S17R37C protofilaments form a symmetric double helix fibril polymorph in 10% of usable particles (Extended Fig. 7). The S17R37C polymorph core spans the residues Y69-Q132, encompassing a longer core region than that in the S17R4C amyloid (Fig. 1b). S17R37C contains six β-strands, with residual weak electron densities observed, likely corresponding to an unidentified peptide on the N-terminal region (Fig. 1f, red arrow). The S17R37C backbone adopts an S-shaped fold centered around a long β-strand spanning the region K102-Q114. Surrounding this central β-strand are the hydrophobic patches (“F92, P94, F104” and “P84, L110”) and the steric zippers composed of “Q80, Y82, Q114”, “Y88, Q91, Y106, N108” and “N107, N105, Q130”), both of which are thought to contribute to stabilizing the core structure. Similar to the S17R4C amyloid, a fuzzy coat was observed on the N-terminal side of the S17R37C polymorph (Extended Fig. 1e, red arrow), although its electron density was weaker than that in S17R4C amyloid. This result suggests that the N-terminal region in S17R37C amyloid is more dynamic and/or less compact than that in S17R4C fibrils. Surprisingly, the minor S17R37N polymorph core comprises the N-terminal residues 7–62, which is completely non-overlapping with the major S17R37C polymorph core (Extended Fig. 8a). Like S17R4N, S17R37N is a right-handed fibril (Extended Fig. 8b). The S17R37N polymorph backbone adopts a winding, S-shaped fold where a central region from N27 to P41 forms zipper-like interactions with surrounding residues (Extended Fig. 8b). The S17R37N polymorph has two solvent-accessible pockets within the fibril core, surrounded by G31, G51, Q50, S53, and Y32, A37, Q38, Y45, and Q47, respectively, with tubular densities present, possibly representing water or other solvent molecules. The S17R37N polymorph core is composed of almost entirely polar residues (Extended Fig. 8b). Furthermore, unlike the S17R4C and S17R37C polymorphs, the S17R37N polymorph contains an ordered R17, located on the solvent-exposed surface of the fibril (Fig. 1e,f; Extended Fig. 8b).

### Comparison of Sup35NM fibril structures

**Figure 2:**
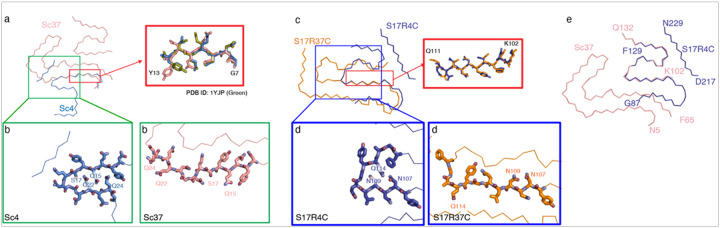
Comparison of the Amyloid Structures Formed by Sup35 **a**, Structural comparison of WT Sup35. The Sc4 structure is shown in blue, and Sc37 in red. Although the cores differ significantly in both size and shape, residues 4–19 closely overlap. This region includes the ^7^GNNQQNY^13^ sequence. The stick models showing superposition of the GNNQQNY segment from the cryo-EM structures of Sc4 (cyan) and Sc37 (pink) with the crystal structure (green, PDB ID: 1yjp). **b**, b-arch formed by residues 13–25 in Sc4 (left). Q15, Q22, and Q24 form a densely packed steric zipper. In Sc37 (right), this region is extended, disrupting the steric zipper. **c**, Structural comparison of S17R amyloids. S17R4C is shown in blue, and S17R37C in orange. Although they share most of the same core-forming region, only residues 102–111 show a high degree of structural similarity. S17R4 exhibits a more compact fold overall. **d**, Close-up view of residues 106–117 in S17R4C (left). In S17R37C (right), this region is extended, whereas in S17R4, N107 and Q114 it forms a hydrogen bond, producing a more compact fold. **e**, Comparison of Sc37 (pink) and S17R4C (blue). The subunit 2 region of Sc37, which spans the PrD-C segment, closely resembles the main core of S17R4C across a broad region (residues 110–130).

Sc4 and Sc37 both form their fibril cores using PrD-N residues, with N5-Y35 being common in both fibrils. Residues N5-Q18 adopt a similar conformation in both fibrils (Fig. 1b); however, a divergence in the backbone direction at residue Q18 results in Sc4 and Sc37 adopting significantly different structures with minimal similarity (Fig. 2a,b). Interestingly, the structure of residues G7-Y13 in Sc4 and Sc37 closely resemble that of the ^7^GNNQQNY^13^ peptide crystals determined by X-ray crystallography^20^. Given that structurally distinct Sc4 and Sc37 fibrils are formed from the same sequence, they can be termed “packing” polymorphs^21,22^. The Sc4 and Sc37 packing polymorphism is likely a result of the difference in aggregation temperature. Nonetheless, conservation of the structure of segment N5-Q18 in the two polymorphs suggests that this segment drives de novo formation of both Sc4 and Sc37 fibrils.

The cores regions of S17R4C and S17R37C fibrils both encompass a common residue range (87–129) from PrD-C (Fig. 1b), and the backbone structures spanning the residues K102-Q111 are similar (Fig. 2c). Nonetheless, S17R4C and S17R37C display significantly different conformations in the residues G112-Q114 (Fig. 2c, d), likely owing to their different aggregation temperatures, making them packing polymorphs. Comparing the WT and the S17R4C/S17R37C mutant fibrils highlights the starkly different, almost non-overlapping residue ranges found in the fibril cores (WT: PrD-N; S17R4C/S17R37C: PrD-C). Intriguingly however, the residues N103-Q130 of subunit 2 in Sc37 and S17R4 amyloids share a similar backbone structure despite Sc37 and S17R4 fibrils being formed at different temperatures and with one amino acid difference (Fig. 2e).

Unexpectedly, both S17R4N and S17R37N form cores using PrD-N (S17R4N: S4-Q38, N108-A115; S17R37N: G7-Q62), unlike S17R4C and S17R37C. However, despite sharing a significant overlapping residue range (G7-Q38), their backbones fold into significantly different conformations, again highlighting the impact of aggregation temperature on fibril conformation (Extended Fig. 9). For instance, while S17R37N has a β-sheet in the N-terminal residues G7-Y13, similar to Sc4 and Sc37, G7-Y13 in S17R4N adopt a kinked conformation in which they participate in an inter-layer hydrogen-bonding network involving the side chains of N5, Q10, Y13 (Extended Fig. 5c). Notably, residues N108-A115 are ordered in S17R4N, connected by a long 70 residue disordered region to Q38, thus giving S17R4N a short ordered residue range that is common with S17R4C and S17R37C (Fig. 1b,e,f; Extended Fig. 5a-b). The PrD-N cores of S17R4N and S17R37N were surprising given that our previous mass spectrometry results only detected PrD-C cores for both S17R4 and S17R37^11^; however, the low abundance of the S17R4N and S17R37N polymorphs may explain their absence in the mass spectrometry data.

### Determination of conformational space of Sup35NM monomer by force spectroscopy

Our cryo-EM structures show that the S17R mutation has a dramatic influence on the residues selected for fibril formation. In the WT Sc4/Sc37 fibrils, the N-terminal core is selected, while in the S17R4/S17R37 fibrils, the PrD-C core is selected in nearly all fibrils except the minor S17R4N and S17R37N polymorphs. The WT Sc4 structure reveals that S17 is tightly packed within the core, rationalizing why the S17R mutation is incompatible with the Sc4 structure (Fig. 2b). However, in the Sc37 structure, S17 is solvent-exposed and could accommodate the S17R mutation (Fig. 2b). Therefore, it is mysterious why the S17R mutant sequence does not adopt the Sc37 structure at 37°C, and instead, the majority of monomers form the S17R37C polymorph using an entirely different region of the protein (PrD-C). Consistent with the structures observed here, our previous mass spectrometry analysis showed that the S17R mutant fibrils seeded by Sc4 fibrils at 4°C and Sc37 fibrils at 37°C adopted the PrD-N (Sc37) core structure^11^. This indicates that the Arg17 in the S17R mutant can be accommodated into the PrD-N core. Indeed, a small fraction of S17R does form a PrD-N core at 37°C in the S17R37N polymorph (Extended Fig. 7, 8). On the other hand, in the presence of S17R fibril seeds, the WT monomer was incorporated into the PrD-C core at both temperatures^11^, indicating that WT monomer is able to form the PrD-C core when the nucleation process is bypassed. These observations indicate that the fibril structures alone cannot explain why and how the WT and the S17R mutant monomers select the PrD-N and PrD-C core, respectively, during *de novo* amyloid formation. Interestingly, our previous NMR analysis suggested that Sup35NM WT might possess compact local structures despite its intrinsically disordered nature whereas the S17R mutant has a different monomer conformation^11^, suggesting that structural differences at the monomer level may influence which residues are available for fibril formation. However, due to the limitation of the ensemble method of NMR spectroscopy, it has remained unclear whether the monomer conformational space (*i.e*. a range of distinct conformations that a monomeric protein can adopt) is different between WT and the S17R mutant.

**Figure 3:**
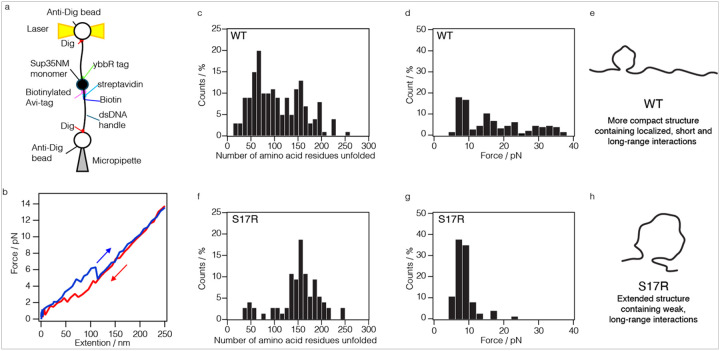
Determination of conformational space of Sup35NM monomer by optical tweezers **a**, Experimental design schematic for force measurement of a single Sup35NM molecule **b**, Force-extension curves show mechanical unfolding (blue) and spontaneous refolding (red) of a single Sup35NM molecule. **c, f,** The distribution of unfolding rip sizes of (**c**) WT or (**f**) S17R mutant derived from force-extension curves was indicated. The X and Y axes indicate the number of amino acid residues unfolded and the ratio of the number of unfolding sizes by stretch of a single molecule, respectively. **d, g,** The distribution of unfolding rip forces of (**d**) WT or (**g**) S17R mutant derived from force-extension curves were indicated. The X and Y axes indicate the number of amino acid residues unfolded and the forces by stretch of a single molecule, respectively. **e**, Schematic of WT monomer conformational space. **h**, Schematic of S17R mutant monomer conformational space.

To investigate the conformational landscape of Sup35NM WT and S17R mutant, we employed force spectroscopy, a single molecule technique. We attempted to mechanically unfold a single molecule of Sup35NM that is tethered by double-stranded DNA handles using optical tweezers^23,24^ (Fig. 3a). To this end, unique Avi and ybbR tags were introduced into the N- and C-terminal sides of Sup35NM, respectively, which were used to attach 2 kilo base pairs of DNA handles that are conjugated at their other end with digoxigenin (Dig). In this way, a single Sup35NM molecule attached by its termini to two DNA handles could be tethered between a bead held in a pipette and a bead held in an optical trap. (Fig. 3a). A single Sup35NM molecule was stretched from 3 to 35 pN by moving the optical trap relative to the pipette, while we monitored both the force and the resulting molecular extension (Fig. 3b). The appearance of a rip in the force-extension curve indicates an unfolding event of Sup35NM, providing the information of the force and the length of amino acid residues that were involved in the mechanical unfolding event^25^. Remarkably, we observed at least one rip in approximately 40% of all the stretching events of Sup35NM WT, although this frequency was expected to be very low due to the disordered nature of Sup35NM protein as evidenced by the circular dichroism (CD) spectrum (Extended Fig. 10). Notably, we detected a single rip in most of the force-extension curves while observation of more than two rips was rare.

To reduce any possible bias of the single-molecule measurements, we typically acquired force-extension curves approximately ten times from one single Sup35NM molecule captured, and this analysis was performed for more than ten distinct single Sup35NM molecules which are derived from three independent sample preparations. Although proteins can spontaneously refold into native states after mechanical unfolding, our observation of rips in the refolding process of Sup35NM was relatively rare. In order to gain more reliable data, therefore, we focused our analysis on the force-extension curves during the unfolding process of Sup35NM. We fitted force-extension curves with a worm-like chain model and obtained scatter plots of the force and the extension from ruptured rips positions upon mechanical unfolding of Sup35NM (Extended Fig. 11). We then analyzed the data depending on the length of extended amino acid residues and the strength of the force (Fig. 3c, d, f, g). Interestingly, we found that Sup35NM WT monomer has various local structures involving 20–200 amino acid residues (Fig. 3c) although the ensemble method of CD spectroscopy showed that Sup35NM protein is overall disordered (Extended Fig. 10).

Next, we performed single molecule measurements of the S17R mutant. Strikingly, the S17R mutation had a dramatically altered monomer conformational space (Fig. 3f–h). In contrast to WT, the S17R mutant showed an increased fraction of the local structure with long-range interactions involving a span of about 150 residues. Notably, the local structures in the S17R mutant monomers were unfolded by weaker forces of 5–10 pN than those in WT monomers, indicating that the local structures in the S17R mutant are formed by dominantly weak, long-range interactions. Together, these findings indicate that the conformational space of Sup35NM monomer is markedly different between WT and the S17R mutant, and that the S17R mutation induced an extended monomer structure with weaker long-range intramolecular interactions, making it easier to unfold. (Fig. 3e, h).

### Amyloid stability through intra- and inter-layer interactions

**Figure 4:**
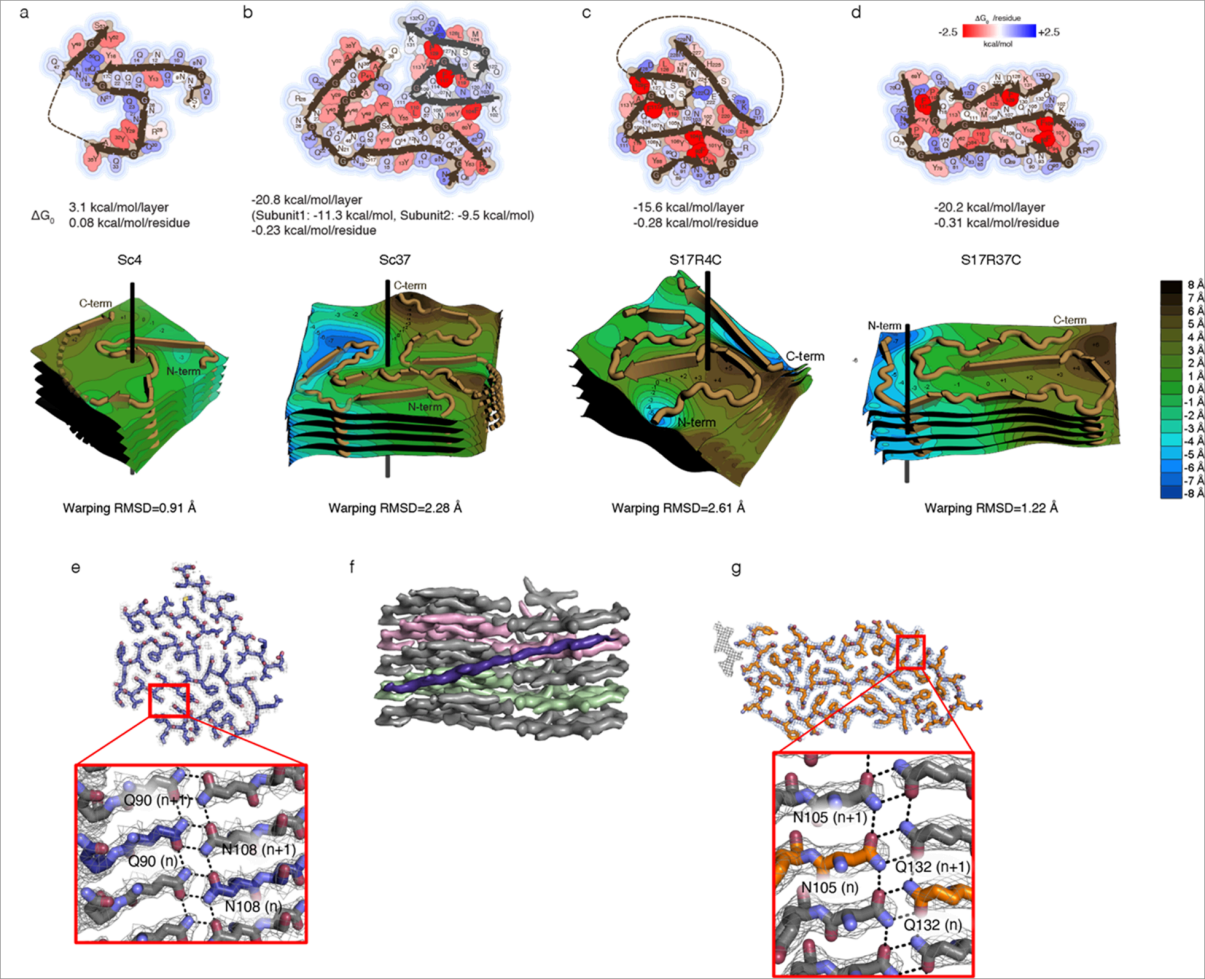
Standard free energy of stabilization of the ordered core of the Sup35NM fibril **a-d**, (Top) Stabilization energy maps of Sup35NM (a) Sc4 (b) Sc37 (c) S17R4C (d) S17R37C amyloids. Each amino acid residue is color-coded according to its stability, with red indicating −2.5 kcal (i.e., more stable) and blue indicating +2.5 kcal (i.e., less stable). (Bottom) Topographical maps of Sup35NM amyloids. **e**, In S17R4C, Q90 (n) and N108 (n+1) form inter-layer interactions, with their side chains offset by precisely one layer. **f**, Additionally, the β-strand consisting of T143-A155 is oriented obliquely relative to the main core layers and spans three layers from its N- to C-terminus. **g**, In S17R37C, N105 (n) and Q132 (n+1) form inter-layer interactions, with their side chains offset by precisely one layer.

To quantify the stability of the fibril structures, we calculated their stabilization energy using the Amyloid Illustrator package^18,26^. Remarkably, the Sc4 amyloid, which is almost entirely composed of polar residues (Fig. 1c, right), has a stabilization energy of + 3.1 kcal/mol per layer. These values are on par with the least stable amyloid structure determined to date (Fig. 4a, Table 2, orb2, PDB ID 6vps, + 3.6 kcal/mol/layer the website of amyloid atlas 2025). The poor stabilization energy can be explained by the fact that intramolecular interactions in each layer primarily involved a small number of residues such as Q15, Q22, and Q24. Furthermore, the interactions along the Sc4 fibril axis are largely mediated by hydrogen bonds between the main chains, with limited contributions from hydrophobic interactions and π-stacking involving aromatic amino acids.

Sc37 amyloid has a significantly higher structural stability than Sc4 fibrils (Fig. 4b, Extended Fig. 12 and Table 2). The stabilization energy per layer was − 11.3 and − 9.5 kcal/mol/subunit for subunit 1 and subunit 2, respectively, indicating that subunit 1 is slightly more stable. Consistent with its greater stability, subunit 1, but not subunit 2, is present in both of the two Sc37 polymorphs observed (Extended Fig. 3). The stabilization energy for the entire fibril (subunits 1 and 2) was − 20.8 kcal/mol/layer. Like Sc4, Sc37 contains many polar residues in its core; however, the side chains in Sc37 form extensive steric zipper interactions that significantly increase buried surface area. Additionally, the Sc37 core has a greater number of stabilizing aromatic and hydrophobic residues compared to Sc4, contributing to its greater stability.

For the four S17R mutant fibrils, S17R4N, S17R4C, S17R37N, S17R37C, the stabilization energies were −0.9, −15.6, −5.0, and − 20.2 kcal/mol per layer, respectively (Fig. 4c,d, Extended Fig. 5d, 8c and Table 2). Interestingly, the more stable polymorphs S17R4C and S17R37C are also the most abundant polymorphs in their respective mixtures (S17R4N:S17R4C = 1:4 and S17R37N:S17R37C = 1:3). A notable feature of the S17R4N and S17R4C polymorphs are their pronounced molecular warping. To quantify this structural property, for each polymorph we calculated the RMSD of Cα atoms within the fibril core relative to a best-fit-plane through one layer of the fibril, which we term the “warping RMSD”^18^. S17R4N and S17R4C have warping RMSDs of 2.82 and 2.61 Å, respectively (Fig. 4c, Extended Fig. 5e, Table 3). For S17R4N, significant elevation changes occur in the N-terminal residues S4-N9 and again in N26-Y32, leading to many inter-layer interactions. Similarly, S17R4C has significant elevation changes that allow complex inter-layer hydrogen bonding, such as that between Q90 (n layer) and N108 (n + 1 layer) (Fig. 4e). Additionally, the long β-strand corresponding to the residues D217-N229, located on the fibril surface rather than within the fibril core, spans the Z-axis direction from the n (green) layer to the n + 2 (pink) layer (Fig. 4f). This additional β-strand likely contributes to the stability of the fibril along the Z-axis. The β-strand is substantially tilted relative to the amyloid axis, suggesting that the extra β-strand might form as a new connection along the Z-axis for fibril stabilization after elongation of the S17R fibrils. Additionally, the residues spanning N105-Q132 in S17R37C form inter-layer hydrogen bonds (Fig. 4g). Although the interactions cause some non-planarity in the layers, the extent of the distortion is limited, with an RMSD of 1.22 Å (Fig. 4d and Table 3). These inter-layer interactions are suggested to enhance the stability of the S17R37 fibrils along the Z-axis, similar to S17R4 fibrils. Notably, the S17R37N polymorph has limited warping (warping RMSD = 1.46 Å), which may contribute to its lower stability (Extended Fig. 8c, d).

## Discussion

In this study, the cryo-EM analysis revealed that the cores of WT Sup35NM fibrils have an almost non-overlapping residue range with S17R4C and S17R37C mutant fibrils (PrD-N, PrD-C, respectively). Additionally, WT and mutant fibrils exhibit distinct amino acid packings and stabilization energies. We found that the stabilization energies of Sc4 and S17R4N (3.1 and −0.9 kcal/mol/layer, respectively) are characteristic of reversible or functional amyloids such as Orb2 and FUS LC domain at 3.6 kcal/mol/layer^22,27–29^. The stabilization energies of Sc37, S17R4C, S17R37N/C fibrils were also comparable to the values reported for reversible amyloids such as FUS (−14.4 kcal/mol/layer^29^; −14.0 kcal/mol/layer^22^; −12.2 kcal/mol/layer^28^) and hnRNPA2 (−21.4 kcal/mol/layer^30^), but much smaller than those of the pathological fibrils from Alzheimer’s disease-associated tau (−68.9 kcal/mol/layer^31^) and multiple system atrophy-associated α-synuclein (Type I: −80.4 kcal/mol/layer; Type II: −73.0 kcal/mol/layer^32^).

Among amyloid fibril structure studies, our experimental system is unique in that it allows us insight into the effect of temperature on fibril structure. In general, the six cryo-EM structures determined here suggest that a lower fibril formation temperature favors more compact fibril structures by reducing the number of residues in the fibril core and producing shorter b-strands. For example, the WT Sc4 fibrils have a small core composed of only 37 residues in which Q15–Q24 (QYSQNGNQQQ) form a compact b-arch, with glutamines densely packed through interactions involving Q15, Q22, and Q24 (Fig. 2b). In contrast, 80 residues comprise the Sc37 core in which residues Q15-Q24 are linearly extended, forming a single b-strand instead of a compact b-arch. Regarding the S17R fibrils, the S17R4C fibril core comprises 54 residues and residues N107–Q114 (NNNLQGYQ) adopt a compactly folded structure while the S17R37C fibril comprises 64 residues with N107–Q114 adopting a linear structure (Fig. 2d). Additionally, S17R4N comprises 43 residues while S17R37N comprises 56. The differences described above may be due to the increased thermal energy at higher fibril formation temperatures allowing residues to escape kinetically trapped conformations, thus leading to more residues in the fibril core and more extended b-strand stretches.

**Figure 5:**
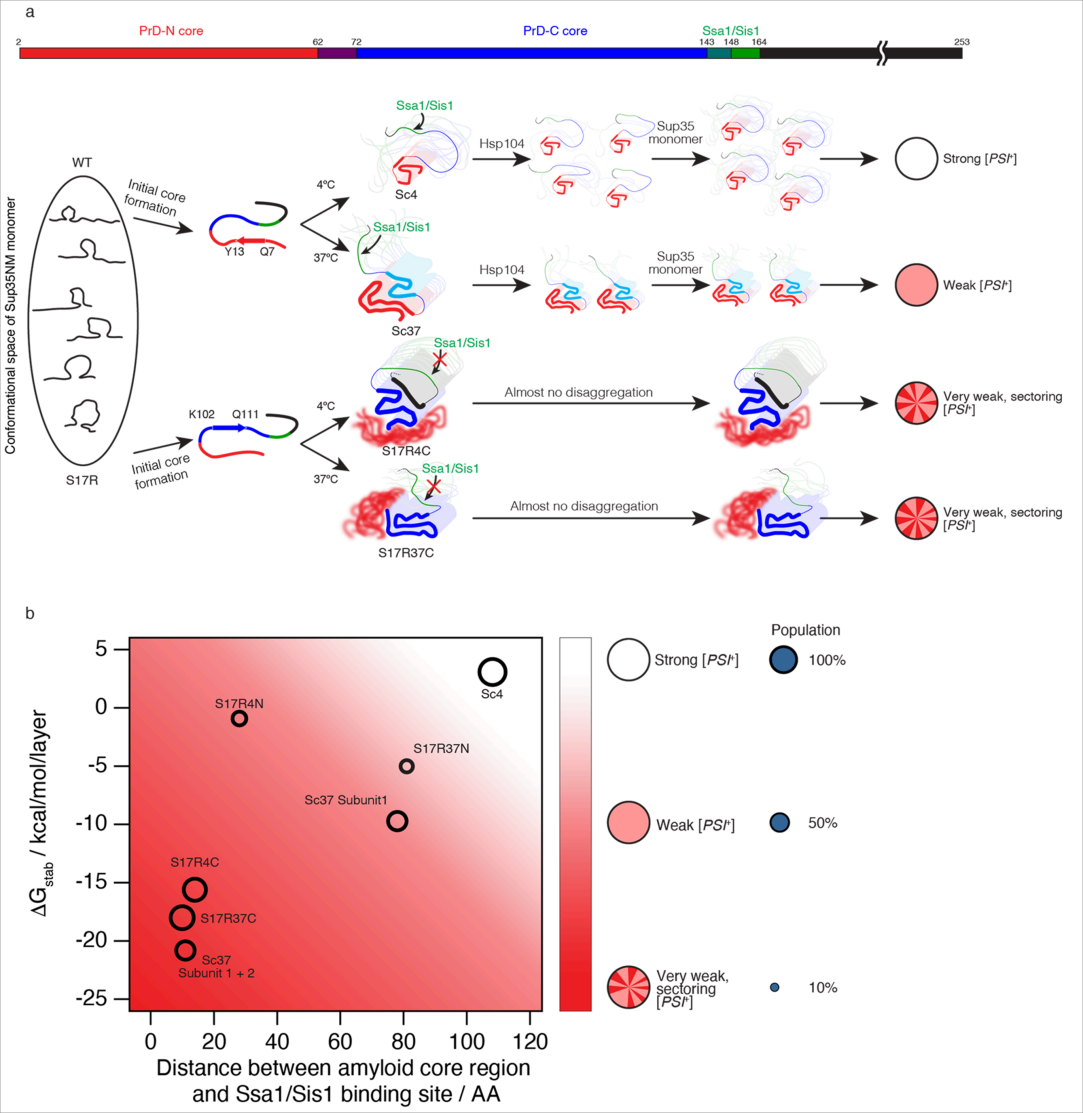
Relationship between Sup35 monomer conformation, amyloid fibril structure, and yeast strain phenotype **a**, Mechanism of amyloid core selection in Sup35NM. Differences in local monomer structures between WT and S17R mutant Sup35NM influence whether the PrD-N or PrD-C core is selected. Subsequently, temperature further influences the fibril core structure that ultimately forms. When a fibril core is formed, an accessibility of Ssa1/Sis1 to the fibril and a disaggregation efficiency are determined. Strength and stability of yeast prion strains is determined by how many seeds (propagons) are yieleded by chaperone-mediated fibril fragmentation. **b**, Relationships between stabilization energy of amyloid core, distance between amyloid core region and the Ssa1/Sis1-binding site, and prion strain phenotypes in yeast. The size of a circle represents a population of the fibril structure among polymorphs. Yeat prion strain phenotypes are shown as a white-red color gradient.

Our single molecule optical tweezers (smOT) analysis helps explain why different regions of the Sup35NM sequence are selected to form the cores of WT and S17R fibrils. Namely, the difference between the more compact, stable interactions in the WT monomer versus the long-range, labile interactions in the S17R monomer likely leads to different residues being available for fibril core formation. Nonetheless, our smOT data cannot explain why certain residues are ordered in particular polymorphs (e.g., PrD-N in Sc4/Sc37 and PrD-C in S17R4C/S17R37C). On one hand, it could be that residues participating in semi-ordered intramolecular interactions in the monomer coalesce into the ordered fibril core. For instance, such residues could be forming intramolecular β-arches in the monomer that correspond to those present in the fibril structures. On the other hand, it is possible that residues participating in intramolecular interactions at the monomer level could be blocked from bonding with additional monomers to nucleate fibril formation. For example, in WT monomer, PrD-C residues might be buried, preventing the assembly of the PrD-C nucleation core (K102–Q111) while the PrD-N nucleation core (G7–Y13) assembles instead in both the Sc4 and Sc37 fibrils (Fig. 5a). In line with this scenario, the strong fuzzy coat present in S17R fibrils, especially S17R4, suggests the N-terminal region that is excluded from the fibril core may be adopting a compact structure (Extended Fig. 1c,e red arrows). Further, the scenario in which residues adopting local structure in the monomer are prevented from assembling into a fibril has been shown to occur for the amyloidogenic VQIVYK sequence in tau protein^33^ However, future work is needed to interrogate the interactions present in Sup35NM monomer in order to fully understand the relationship between the monomer conformational landscape and fibril structure.

The atomic fibril structures, energetic analysis, and our previous work on chaperone-mediated fibril disassembly allow for a structure-based mechanistic understanding of how Sc4, Sc37 and S17R4/S17R37 fibrils, upon infection of [*psi*^−^] yeast, induce strong, weak, and very weak/sectoring [*PSI*^+^] phenotypes, respectively^14^. Sc4 fibrils contain the most compact core of 37 residues and few intra-layer interactions. In fact, the DG value of Sc4 was positive, making the Sc4 fibrils the most energetically unstable amyloids determined to date^18^. In addition, the freely accessible Ssa1/Sis1 binding site (T143-K164) far from the fibril core allows the Sc4 fibrils to be readily fragmented by Hsp104/Ssa1/Sis1 chaperones^14^. Together, the low stability and ready chaperone-mediated fragmentation facilitate the production of many Sc4 fibril fragments (propagons)^12^ that recruit more Sup35 monomers to the fibrils, increasing overall Sup35 aggregates and leading to white, strong [*PSI*^+^(Sc4)] phenotypes (Fig. 5a). By contrast, Sc37 fibrils showed an extended and stable PrD-N core structure involving 61 residues. Additionally, although the Ssa1/Sis1 binding region is far outside the Sc37 subunit 1 core, the partial existence of subunit 2, which neighbors the Ssa1/Sis1 binding region, is likely responsible for the lower susceptibility of Sc37 to chaperone-mediated fragmentation^14^. Together, the greater stability and reduced chaperone-mediated fragmentation help to explain the smaller number of propagons in [*PSI*^+^(Sc37)] yeast and the induction of the non-sectoring pink, weak but stable [*PSI*^+^(Sc37)] phenotypes^9,12^.

Similar to Sc37, S17R4 and S17R37 fibrils have greater stabilities than Sc4 fibrils due to their long fibril cores and extensivepp-stacking, which likely impairs fibril fragmentation. Additionally, the S17R4/S17R37 structures exhibit inter-layer interactions by short peptides, which further reinforces the axial fibril stability. Perhaps most importantly, the PrD-C core regions of the S17R4C and S17R37C fibrils either partially overlap or border on the Ssa1/Sis1-binding site (T143-K164), respectively, which explains the previously observed resistance to chaperone-mediated fragmentation^15^. Alternatively, the S17R37N polymorph has a highly accessible chaperone-binding domain, which should facilitate chaperone-mediated fragmentation and propagation. However, likely due to S17R37N’s low population, the S17R37 fibril preparation (containing S17R37N and S17R37C polymorphs at a 1:4 ratio) still acts as a very weak prion. Interestingly, in S17R4N, although the main fibril core is far away from the chaperone-binding site, residues N108-A115, which are adjacent to the chaperone-binding site, form an ordered b-sheet alongside the fibril core. The ordering of residues adjacent to the chaperone-binding site, along with S17R4N’s low abundance, help explain why the S17R4 fibril preparation (containing S17R4N and S17R4C polymorphs at a ~1:3 ratio) acts as a very weak prion despite S17R4N’s lower stability. Collectively, the higher stability and resistance to chaperone-mediated fragmentation explain the potentially very low amount of propagons in [*PSI*^+^(S17R)] yeast and the induction of characteristic sectoring, very weak [*PSI*^+^] phenotypes.

Previously, we showed that the strength of the prion strain phenotype, which is correlated with the amount of Sup35 aggregates in yeast cells, is dependent on both the rate of fibril elongation and the rate of fibril fragmentation^12^. Further, we demonstrated that the rate of fibril fragmentation, through the production of seeds, has a greater impact on the amount of aggregated Sup35 than the rate of fibril elongation^12^. However, it had remained unclear what factors regulate the rate of fibril fragmentation. Our data in this study suggest that two major factors, the fibril stabilization energy and the distance between fibril core residues and the Ssa1/Sis1 binding region (D143-T164), determine the fibril fragmentation rate. We illustrate this relationship in a theoretical structure-phenotype landscape in Fig. 5b where fibrils with lower stability and a greater distance between fibril core residues and the exposed Ssa1/Sis1 binding site lead to the strongest [*PSI*^+^] strain phenotypes. These phenotypes result from the fact that less stable fibrils are more susceptible to fragmentation into seeds, through mechanical and chaperone-mediated means, thus leading to stronger [*PSI*^+^] strain phenotypes. In a similar vein, since Ssa1/Sis1 binding to the region D143-T164, which is highly conserved in yeast species (Extended Fig. 13), triggers fibril fragmentation by Hsp104^14^, a greater distance between the fibril core region and the Ssa1/Sis1 binding region increases chaperone accessibility, chaperone-mediated fragmentation, the production of seeds, and thus stronger [*PSI*^+^] strain phenotypes (Fig. 5b). The six fibril structures we determined here are plotted on the graph in Fig. 5b to demonstrate the relationship between fibril stability, chaperone accessibility, and strain phenotype. However, we note that the graph in Fig. 5b is speculative and our current set of Sup35 fibril structures only explore a very limited area of the structure-phenotype landscape. Future work determining the phenotype and structure of a greater variety of fibrils occupying different areas of this landscape (e.g., unstable fibrils with low chaperone accessibility) would be needed to further strengthen our hypothesis. Of note, we include two data points representing Sc37 fibrils: one including only subunit 1, which induces lighter pink color phenotypes (star) and another which includes both subunits 1 and 2, which elicit dark pink, unstable phenotypes (diamond). However, since these two polymorphs co-exist in the Sc37 fibril preparation, we are not able to assign at present their exact phenotypes experimentally.

Notably, this study solves previous otherwise puzzling observations in vivo. First, previous studies reported that N100 and N109 are critical to formation of the PrD-C amyloid core^11^, and yeast genetic analysis with wild type yeast strains indicates that N109 plays important roles in Sup35 prion transmission^34^. We found that N100 is in the ordered core in S17R4C and S17R37C while N109 is in the ordered core of S17R4N, S17R4C, and S17R37C. Thus, the cryo-EM structures explain why the N100A/N109A mutations in the S17R fibrils disrupt the PrD-C core, leading instead to formation of the PrD-N core^11^. Second, the previous genetic screening analysis for Sup35NM mutations revealed the formation of anti-suppressor, weak [*PSI*^+^] yeast by the selective Q15R, S17R, Q22R, or Q24R (but not Y16, N21, and Q23) mutation albeit by unknown mechanisms^17^. Remarkably, all the Q15, S17, Q22, and Q24 residues form a steric zipper and are buried in the Sc4 structure (Fig. 2b), implying that the mutations inhibit fibril elongation, ultimately leading to weak [*PSI*^+^] phenotypes. Alternatively, Y16, N21 and Q23 residues in Sc4 fibrils are all solvent-exposed, allowing them to accommodate mutations without disrupting the Sc4 fibril conformation.

This study provides mechanistic insight into propagation and pathophysiology of amyloid fibrils in neurodegenerative diseases such as synucleinopathies and tauopathies. A recent study proposes that DNAJB1 binds outside the core of a-synuclein fibrils and recruits Hsc70 to disassemble the fibrils^35^. Notably, this mechanism is similar to that of the Hsp104/Ssa1/Sis1-mediated disaggregation of Sup35NM fibrils studied here, where Ssa1/Sis1 first bind to the fibrils, followed by Hsp104-binding^14^ Furthermore, Jäger, *et al*., recently showed that a-synuclein fibril polymorphism likely determines the effectiveness and mode of Hsc70/DNAJB1 chaperone-mediated disaggregation^36^. Both of these studies suggest that the relationship between amyloid fibril structure, chaperone activity, and seed production that we have outlined here for yeast prion strains may explain the structure-phenotype relationship observed in synucleinopathies, tauopathies, and other neurodegenerative diseases. However, to date, our study is the only one to holistically connect fibril atomic structure, chaperone disaggregation activity, and phenotypic outcome in a native biological environment. Thus, further studies leveraging fibril polymorph atomic structure and realistic biological models are needed for synucleinopathies and tauopathies. Further, elucidation of a conformational landscape of intrinsically disordered a-synuclein and tau monomers will also help to reveal the origin of distinct fibril structures in synucleinopathies and tauopathies^37,38^.

## Methods

### Plasmid construction

For bacterial expression of Sup35NM, a pET29b vector including a C terminal 7x histidine-tag was used. Mutations were introduced by site-directed mutagenesis (Takara) and confirmed by DNA sequencing (ThermoFisher).

### Protein purification

Sup35NM proteins have a 7x histidine tag in the carboxyl terminus. The Sup35NM protein was purified by a Ni-NTA column (Qiagen) under the denatured condition, followed by further purification with SOURCE 15S column chromatography (Cytiva) with linear gradient 0 – 1 M NaCl condition using AKTA instrument (Cytiva) as described previously^9,40^. The sample were fractionated by reverse-phase chromatography with C18 column (Protein-R, COSMOSIL) and lyophilized.

### Sup35NM amyloid formation

The first generation (G1) amyloid was formed spontaneously in buffer C (5 mM KPi buffer, 150 mM NaCl, pH 7.4) with mild agitation at 8 rpm (Labquake, Theromo Fisher Scientific) for 24 h. The second generation (G2) of amyloid was formed by polymerization of Sup35NM in the presence of sonicated G1 amyloid (5%, mol/mol) without agitation. The G2 amyloid core was investigated by MALDI. The MALDI was analyzed using the 4800 Plus MALDI-TOF/TOF analyzer (Applied Biosystems) at the Support Unit for Bio-Material Analysis in RIKEN Center for Brain Science, Research Resources Division (RRD). The G2 amyloid was then sonicated and flash frozen by liquid nitrogen as G2 seeds. The G3 amyloid were generated in the presence of G2 seeds (5%, mol/mol), as above.

### Cryo-EM data collection and processing

The G3 amyloid were collected by centrifugation at 30000 × g for 10 min at room temperature, followed by removal of 90% (vol/vol) of the supernatant. 2 ml of Sc4 and Sc37 fibril solutions were applied to glow-discharged Quantifoil 1.2/1.3 electron microscope grids and plunge-frozen into liquid ethane using a Vitrobot Mark IV (Thermo Fisher Scientific). S17R4 was prepared by adding F300F (Anatrace) at a final concentration of 2.8 mM, depositing 3 ml onto a Quantifoil 1.2/1.3 electron microscope grid, and plunge-freezing using a Vitrobot. S17R37 was prepared by depositing 3 ml onto a graphene-coated grid and plunge-freezing with a Vitrobot.

Sc4 data were collected on a Titan Krios (Thermo Fisher Scientific) microscope located at the Stanford-SLAC Cryo-EM Center equipped with a BioQuantum/K3 setup (operated with 300 kV acceleration voltage and slit width of 20 eV) and automated with EPU (Thermo Fisher Scientific). Counting mode movies were collected with a nominal physical pixel size of 1.078 Å per pixel and a dose per frame of 1.5 e^−^/Å^2^. A total of 27 frames were taken for each movie, resulting in a final dose 40.5 e^−^/Å^2^ per image. Sc37 data were taken on a similar Titan Krios/BioQuantum/K3 setup at the UCLA Electron Imaging Center for Nanosystems, automated with SerialEM^41^. Super-resolution movies were collected with a calibrated pixel size of 1.078 Å/pixel (0.539 Å/pixel in super-resolution movie frames) and a dose per frame of ~1.25 e-/Å^2^. A total of 40 frames were taken for each movie, resulting in a final dose of ~50 e-/Å^2^ per image.

For Sc4 and Sc37 datasets, cryo-EM movies were motion corrected in RELION^42^ and CTF estimation was performed using CTFFIND4^43^. All particle picking was performed in crYOLO^44^. For both Sc4 and Sc37 datasets, a subset of ~100 images were used to manually select fibrils, train the model, and subsequently perform automated fibril picking on the data sets. Classification, helical reconstruction and three-dimensional (3D) refinement were performed in RELION as described^45^. 2D classifications of 1,024-pixel boxes were used to estimate helical parameters. We performed 3D classification with the estimated helical parameters for fibrils and an elongated Gaussian blob as an initial model to generate starting reconstructions. We ran additional 3D classifications using the preliminary reconstructions from the previous step to select for particles contributing to homogenous classes. Refine3D was used for auto-refinement to achieve the final reconstructions. We performed the map–map Fourier shell correlation (FSC) with a generous, soft-edged solvent mask and high-resolution noise substitution in RELION PostProcess, resulting in a resolution estimate of 3.3 Å for Sc4 and 3.1 Å for Sc37. To determine that Sc37 fibrils contain both particles with and without subunit 2, we analyzed the particles.star file after 3D classification and calculated the percentage of particles with and without subunit 2 that are assigned to the same tube ID.

S17R4 and S17R37 were imaged on a Titan Krios G4 operating at 300 kV equipped with a K3 direct electron detector (Gatan) and a Bioquantum energy filter with a slit width of 15 eV at a magnification of 105,000×, resulting in a pixel size of 0.83 Å, using EPU software (Thermo Fisher Scientific). These data were collected at the RIKEN Yokohama cryo-EM facility. Data were collected with an exposure time of 2.4 seconds (48 frames) per image, resulting in a final dose of 50 e^−^/Å^2^. Motion-correction and CTF estimation were performed as above. Filaments were picked using crYOLO^44^ with the filaments general model which we have established from multiple amyloid fibrils and extracted at box sizes of 800 and 400 pixels. Subsequent analysis was performed in RELION 5, using the 800-pixel extractions for 2D classification to identify polymorphs with the RELION filament tool^39^. Then, high-quality classes were selected and the crossover distance was determined. An initial model was generated with relion_helix_ inimodel2d^46^and further refined using Refine3D; for S17R4, the Blush regularization^47^ option was applied during refinement. The standard post-processing procedure in RELION yielded resolution estimates of 2.2 Å for S17R4C, 2.4 Å for S17R4N, 2.4 Å for S17R37C and 2.4 Å for S17R37N.

### Atomic model building

We sharpened Sc4 and Sc37 reconstructions using phenix.auto_sharpen^48^ and S17R4N, S17R4C, S17R37N, and S17R37C using RELION post-process at the resolution cut-off indicated by the map–map FSC and subsequently built atomic models into the refined maps with Coot^49^. We performed real-space refinement and comprehensive structure validation of all our final models in Phenix^50^.

### Structural Analysis

PyMol (Schrödinger) was used to make structure figures. Energetic analysis was performed using the program accessiblesurfacearea_v07.2t.f from Amyloid Illustrator package^18,26^. Warping figures and analysis were created using programs obtained from Michael Sawaya^18^.

### Single-molecule optical tweezers experiment

A single Sup35NM monomeric protein was bound to beads through 2,000 bp of dsDNA handles, as previously described^51^. Avi-tag and ybbR-tag were introduced at N terminus and C terminus of Sup35NM, respectively. To biotinylate the Avi-tag, BirA sequence was introduced in Sup35NM plasmid. Therefore, BirA and Sup35NM with Avi-tag and ybbR-tag were co-expressed in BL21 competent cells. To attach the DNA handle to ybbR-tag, we used the reaction of CoA and ybbR tag by Sfp enzyme^51^. A 24 nt long oligopeptide with CoA and 28 nt long oligopeptide with complementary sequence and a 4 nt long single stranded overhang sequence for ligation were annealed. The CoA-dsDNA and Sup35NM with ybbR tag were covalently coupled by Sfp enzyme. The 2,000 bp DNA handles were synthesized by PCR using Taq DNA polymerase (NEB). The forward primer was modified with digoxigenin (Dig) and the reverse primer was modified with Biotin for N-terminal biotinylated Avi-tag. For C-terminal ybbR-tag, the forward primer was modified with Dig and the reverse primer has a restriction enzyme site for ligation of CoA-dsDNA. The PCR products (dsDNA) were purified by QIAquick PCR purification kit (Qiagen). The Sup35NM-dsDNA complex was ligated with digested dsDNA handle at 16°C for 1 hour. After ligation of Sup35NM and DNA handle, it was incubated with 2.1 mm of anti-Dig beads (Spherotech) for 30 min on ice (sample beads). The dsDNA handle with Biotin and Dig was incubated with streptavidin (NEB) and anti-Dig beads for 30 min on ice (handle beads). The sample beads and handle beads were injected to each channel in the optical tweezers chamber. A handle bead was fixed with a micropipette by suction. A sample bead was captured by the optical trap and tethered close to the handle beads. The force measurements were carried out in potassium phosphate buffer (5 mM potassium phosphate, 150 mM NaCl, pH7.5). The single molecule of Sup35NM was stretched and relaxed multiple times between 2 pN and 35 pN by moving the trap (100 nm /sec) relative to the optical trap to obtain force-extension curves. The interval time between measurements was typically about 7 sec at 2 pN to allow the protein to form structures^51–53^. Rupture forces and extension changes from force-extension curves were determined as previously described^52^. To reduce any possible bias of the measurement, we typically acquired force-extension curves approximately ten times from one single Sup35NM molecule captured, and this analysis was performed for more than ten distinct single Sup35NM molecules which are derived from at least three independent sample preparations. The number of amino acid residues stretched by mechanical unfolding was calculated by using a worm-like chain model, as previously reported^52,54^.

## Supplementary Material

Supplementary Files

This is a list of supplementary files associated with this preprint. Click to download.

- ExtendedFigs.docx

## Figures and Tables

**Table 1:** Cryo-EM map and model statistics

	Sc4	Sc37	S17R4C	S17R4N	S17R37C(job202)	S17R37N
	(EMDB-66706) (PDB 9XBN)	(EMDB-66707) (PDB 9xbo)	(EMDB-66708) (PDB 9XBP)	(EMDB-66703) (PDB 9XBK)	(EMDB-66705) (PDB 9XBM)	(EMDB-66704) (PDB 9XBL)
Magnification	81,000	81,000	105,000	105,000	105,000	105,000
Voltage (kV)	300	300	300	300	300	300
Electron exposure (e-/Å^2)	40.5	50.0	50.2	50.2	56.0	56.0
Defocus range (μm)	−1.0 – −2.2	−1.0 – −2.2	−0.6 – −2.0	−0.6 – −2.0	−0.6 – −2.0	−0.6 – −2.0
Pixel size (Å)	1.078	1.078	0.83	0.83	0.83	0.83
Symmetry imposed	C1	C1	C1	C1	C1	C1
Helical rise (Å)	4.83	4.81	4.76	4.79	4.83	4.86
Helical twist (°)	−1.82	−1.31	−3.96	3.95	−1.77	1.50
Initial particle images (no.)	197,751	303,188	4,062,295	4,062,295	2,373,148	2,373,148
Final particle images (no.)	86,542	68,860	655,005	248,947	454,693	120,844
Map resolution (Å)	3.1	3.1	2.2	2.4	2.4	2.4
FSC threshold	0.143	0.143	0.143	0.143	0.143	0.143
Map resolution rage	200 – 3.1	200 – 3.1	200 – 2.2	200 – 2.4	200 – 2.5	200 – 2.4
Initial model used	De novo	De novo	De novo	De novo	De novo	De novo
Model resolution (Å)	3.3	3.3	2.2	2.3	2.3	2.4
FSC threshold	0.5	0.5	0.5	0.5	0.5	0.5
Model resolution	Not applicable	Not applicable	Not applicable	Not applicable	Not applicable	Not applicable
range (Å)						
Map sharpening B factor (Å^2)	−100	−100	−59.0782	−49.8854	−61.1329	−45.9651
Nonhydrogen atoms	1,635	3,770	2,275	1785	2685	2,305
Protein residues	195	460	280	215	325	280
B factors (Å^2)						
Protein	76.15	62.56	31.26	43.22	30.13	39.55
Bond lengths (Å)	0.005	0.003	0.002	0.007	0.004	0.005
Bond angles (°)	0.851	0.699	0.507	0.914	0.739	1.158
MolProbity score	1.95	1.61	1.45	1.77	1.88	1.07
Clashscore	8.08	10.62	4.02	7.59	7.18	2.84
Favored (%)	91.43	97.73	96.15	94.87	92.06	98.15
Allowed (%)	8.57	2.27	3.85	5.13	7.94	1.85

**Table 2:** Stabilization energies of Sup35NM fibril polymorphs.

	ΔG_stab_ kcal/mol/chain	ΔG_stab_ kcal/mol/residue
Sc4	3.1	0.08
Sc37	−20.8	−0.23
Sc37 subunit 1	−11.3	−0.19
Sc37 subunit 2	−9.5	−0.31
Sc37 subunit 1 alone	−9.7	−0.16
Sc37 subunit 2 alone	−7.3	−0.23
S17R4N	−0.9	−0.02
S17R4C	−15.6	−0.28
S17R37N	−5.0	−0.09
S17R37C	−20.2	−0.31

**Table 3:** Warping RMSD of Sup35NM fibril polymorphs.

	RMSD from fibril normal plane (Å)	RMSD from best-fit-plane (Å)
Sc4	1.54	0.91
Sc37	3.27	2.28
S17R4N	2.90	2.82
S17R4C	3.06	2.61
S17R37N	2.13	1.46
S17R37C	2.98	1.22

## Data Availability

All coordinates were deposited in the PDB with the following accession codes: Sc4 (PDBID: 9XBN; EMDB: 66706), Sc37 (PDBID: 9XBO; EMDB: 66707), S17R4N (PDBID: 9XBK; EMDB: 66703), S17R4C (PDBID: 9XBP; EMDB: 66708), S17R37N (PDBID: 9XBL; EMDB: 66704), S17R37C (PDBID: 9XBM; EMDB: 66705).
